# A Structured Quality Improvement Framework for Managing Serratia marcescens Outbreaks in a Resource-Limited Neonatal Intensive Care Unit

**DOI:** 10.7759/cureus.102156

**Published:** 2026-01-23

**Authors:** Mahmoud Khaild, Nasser Al Shafouri, Amna Al Nassri

**Affiliations:** 1 Pediatrics and Neonatology, Ibri Hospital, Ibri, OMN; 2 Infectious Diseases, Ibri Hospital, Al Dhahera, OMN

**Keywords:** infection control, neonatal intensive care, outbreak management, quality improvement, resource-limited settings, serratia marcescens

## Abstract

Background: Neonatal intensive care units (NICUs) in resource-limited settings face major infection-control challenges due to patient vulnerability, high device use, and limited staffing. *Serratia marcescens* is a well-recognized cause of recurrent NICU outbreaks, however structured quality-improvement (QI) frameworks for outbreak management in low-resource settings remain insufficiently described.

Objective: To evaluate whether implementation of a structured, multimodal, Plan-Do-Study-Act (PDSA)-based QI framework was associated with improved outbreak containment and infection-control process measures in a resource-limited NICU.

Methods: We conducted a single-centre, retrospective pre-post quality improvement evaluation comparing two *Serratia marcescens* outbreaks (2022 and 2023) in the neonatal intensive care unit of Ibri Hospital, Oman. The 2023 event implemented a structured Plan-Do-Study-Act (PDSA) framework featuring early-warning triggers, multidisciplinary task-force activation, enhanced staffing, environmental decontamination, and real-time auditing. Primary outcomes were outbreak duration and incidence density (cases per 1,000 patient-days). Secondary measures included compliance with hand hygiene, personal protective equipment (PPE), and environmental hygiene.

Results: Outbreak incidence was observed to decrease from 8.9 to 2.5 cases per 1,000 patient-days, and outbreak duration was shorter, declining from 53 to 14 days. Hand-hygiene compliance improved from 78.9% to 92.0%, environmental-cleaning scores from 78% to 96%, and PPE compliance reached 100%.

Conclusion: Implementation of a structured, PDSA-based outbreak-management framework was temporally associated with improvements in infection-control process measures in a resource-limited NICU. Despite a limited sample size and an uncontrolled pre-post design, this model supports feasibility and measurable benefit in low-resource NICUs.

## Introduction

Neonatal intensive care units (NICUs) care for highly vulnerable infants, preterm and low-birth-weight neonates with underdeveloped immune systems requiring invasive devices such as central lines and mechanical ventilation [1,2]. These factors, combined with high patient density, increase risks of healthcare-associated infections (HAIs) and multidrug-resistant organism (MDRO) transmission [3,4]. In resource-limited NICU settings, these risks are further amplified by constrained staffing ratios, limited isolation capacity, overcrowding, variable access to infection-prevention resources, and reduced availability of real-time surveillance and microbiological support [5-8].

NICU outbreaks have been reported with a wide range of organisms, including *Klebsiella pneumoniae, Acinetobacter baumannii, Escherichia coli, *and coagulase-negative *Staphylococcus *species [3,4,9]. *Serratia marcescens* has become a recurring cause of NICU outbreaks worldwide [10-12]. This opportunistic Gram-negative bacillus can persist on dry surfaces, in disinfectants, and within sink drains, complicating eradication efforts [13]. Neonatal infections range from colonization to sepsis and meningitis, with mortality rates of up to 40% in severe outbreaks [14,15].

Previous studies identified risk factors including low birth weight, prolonged hospitalization, and hand-hygiene lapses [16-18]. However, few interventional frameworks for structured outbreak management have been published from low-resource NICU settings [5,6].

Quality improvement (QI) methodologies provide structured approaches for addressing complex healthcare challenges such as infection prevention and outbreak control. The Plan-Do-Study-Act (PDSA) cycle is particularly suited to outbreak settings because it supports rapid-cycle testing, real-time feedback, and iterative adaptation to local context [19-21]. In contrast, resource-intensive approaches such as Lean Six Sigma or Failure Mode and Effects Analysis may be less feasible during acute outbreaks in resource-constrained NICUs [5,6].

In our setting, a NICU outbreak in 2021 prompted the development of a proactive infection-control model focusing on early-warning triggers, multidisciplinary coordination, and continuous audit. The model underwent iterative refinement through successive Plan-Do-Study-Act (PDSA) cycles, with partial implementation and local adaptation during routine practice in 2022.

An outbreak occurring in 2022 at Ibri Hospital NICU was therefore managed while the framework was still in development and not fully operationalized, relying predominantly on conventional reactive measures such as case-based isolation, routine environmental cleaning, and intermittent infection-control practitioner involvement. By 2023, the model had matured into a fully implemented, structured PDSA-based quality-improvement framework. The 2023 outbreak response thus represents the first comprehensive application of this structured framework, incorporating predefined activation triggers, rapid multidisciplinary task-force mobilization, locally standardized infection-prevention bundles, and real-time audit with feedback.

The objective of this study was to evaluate whether implementation of this structured, PDSA-based, multi-modal infection-control framework was associated with improved outbreak containment and key compliance indicators during *Serratia marcescens* outbreaks in a resource-limited NICU, by comparing outcomes before and after full framework implementation.

## Materials and methods

Study design and setting

This study was a retrospective pre-post quality-improvement (QI) evaluation conducted in the 15-bed Level III NICU at Ibri Hospital, Oman, which admits approximately 480 neonates annually. The evaluation compared two discrete *Serratia marcescens* outbreak events occurring in the same clinical setting but managed using different infection-control approaches. The pre-post design was selected to assess changes in outbreak characteristics and process measures following implementation of a structured QI framework, consistent with improvement science methodology [19,22].

An outbreak was defined as the occurrence of two or more *S. marcescens* cases with epidemiological linkage within the NICU. Outbreak duration was defined as the interval from identification of the first confirmed case to 14 days after the last identified case, in accordance with established outbreak investigation guidance [23].

The first outbreak occurred between 27 April 2022 and 20 June 2022 and was managed using conventional reactive infection-control measures. The second outbreak occurred between 12 September 2023 and 26 September 2023 and was managed following full implementation of a structured PDSA-based quality-improvement framework. The study is reported in accordance with SQUIRE 2.0 guidelines [22].

Study population and sampling

All neonates admitted to the NICU during each outbreak period who met the outbreak case definition were included. Sampling was consecutive and exhaustive; no sampling or randomization was performed. No a priori sample-size calculation was undertaken, as this evaluation included all confirmed cases occurring during the defined outbreak periods.

Data sources

Data were collected retrospectively from microbiology laboratory records, infection-control surveillance logs, staffing records, audit tools, and clinical documentation routinely maintained during outbreak management. No additional data collection was done beyond routine documentation.

Conventional outbreak management (2022)

During the 2022 outbreak, management followed conventional reactive measures. Standard contact precautions (gown and gloves) were applied, and routine daily environmental cleaning was performed. Isolation was implemented on a case-by-case basis depending on space availability. Nurse-to-patient ratios remained at the usual 1:3-4, and infection control practitioner involvement was intermittent. Communication, staffing modifications, and intensified cleaning practices were informal and inconsistent, which limited structured response coordination.

Structured QI intervention framework (2023)

**Figure 1:**
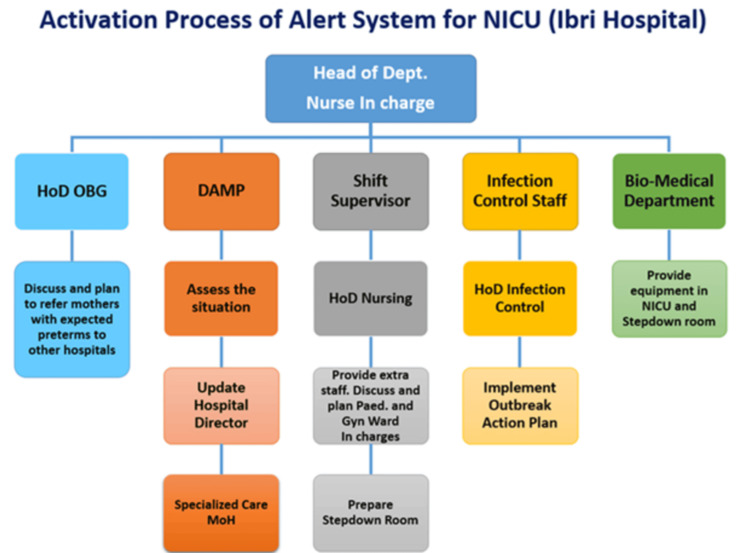
Activation Process of the Alert System for the Neonatal Intensive Care Unit, Ibri Hospital. This flowchart illustrates the escalation pathway for activating the outbreak alert system in the NICU. The process begins with early recognition by the Head of Department and Nurse-in-Charge, followed by coordinated actions across obstetrics, administration, infection control, and biomedical teams to ensure rapid containment, communication, and resource mobilization. HoD: head of department; OBG: obstetrics and gynaecology; DAMP: director of administration and medical planning; MoH: ministry of health; NICU: neonatal intensive care unit.

In contrast, the 2023 outbreak was managed using a structured intervention framework developed through iterative PDSA cycles [19,21]. A pre-alert and alert system was used, with early-warning triggers that included any MDRO case detected, bed occupancy greater than 90% or more than four ventilated infants, staffing below standards (nurse ratio >1:3), and a cluster of unexplained sepsis (≥2 cases within 7 days). These thresholds were selected pragmatically based on local operational constraints and published guidance linking crowding, device burden, and staffing pressure to increased outbreak risk and IPC performance in neonatal settings [5-8]. Alert activation mobilized the outbreak task force within 12 hours (Figure 1).

Core intervention bundles, encompassing case management, environmental controls, staffing modifications, education and training, and communication strategies, were applied (Table 1).

**Table 1:** Multidisciplinary Outbreak Response Checklist for NICU (Ibri Hospital). This checklist outlines key outbreak-response tasks categorized under alert flow, staffing, monitoring, audit, training, and feedback. It defines responsibilities, timelines, and accountability across nursing, infection control, and administrative units to ensure structured, real-time outbreak management. IPC: infection prevention and control; HoD: head of department; AST: antimicrobial stewardship team; PRO: public relations officer; SOS: save our souls, as required/urgent; MoH: ministry of health, MDRO: multidrug-resistant organism.

#	Task	Responsible Staff	Time Frame	Yes	No	In Process
1	Alerts and reporting of new MDRO cases	Lab to IPC; lab to unit staff	ASAP			
2	To update the admin when the outbreak	HoD IPC/nursing	ASAP			
3	Set up an outbreak control team when an outbreak occurs	Admin	SOS			
4	Communication and collaboration between teams	Admin	SOS			
5	Isolation and transmission-based precautions (contact, droplet, airborne or in combination)	IPC, unit staff; nursing supervisor	ASAP/on alert			
6	To prepare a line list	IPC	Daily/SOS			
7	To provide sufficient nursing staff based on clinical care and the number of MDRO cases	Nursing HoD	Daily/SOS			
8	To keep sufficient physicians on the floor	HoD	Daily/SOS			
9	Provide dedicated infection control staff on the floor	HoD IPC	Daily/SOS			
10	Prospective surveillance of MDRO by screening neonates and new admissions	Resident physician on the advice of IPC	Daily/SOS			
11	Hand hygiene compliance and audit	IPC	Daily			
12	Monitoring proper PPE use for each contact	IPC	Daily			
13	Ensure nursing staff stay in allocated rooms	Incharge and IPC	Daily			
14	Environment screening when required	IPC	SOS			
15	Terminal cleaning and fumigation on discharge of cases when rooms are vacant	Unit incharge	SOS			
16	Cleaning and disinfection of shared equipment after every use	IPC	Daily/SOS			
17	Antimicrobial Stewardship audit	AST	Daily/SOS			
18	Monitor attendant control	Unit incharge/PRO	Daily/SOS			
19	Education and training on infection control practices	IPC	Daily/SOS			
20	Education and training on cleaning and disinfection	IPC	Daily/SOS			
21	Education and training of family members	IPC and unit staff	Daily/SOS			
22	Final report on end of outbreak with lessons learned and recommendations to Hospital Admin.	IPC and unit head	SOS			
23	Dissemination of the final report in the form of a CME to other colleagues	IPC and unit head	SOS			

Case management and infection control measures

Case management included immediate isolation and cohorting within four hours, enhanced contact precautions (gown, gloves, mask), dedicated equipment for isolated patients, weekly surveillance cultures with additional targeted screening, and visitor restrictions allowing parents only with mandatory hand hygiene and gown use.

Environmental and staffing interventions

Environmental controls enforced physical spacing of ≥1.2 m between incubators, enhanced cleaning twice daily using 0.5% sodium hypochlorite, high-touch surface disinfection every 4 hours, sink management protocols, creation of a step-down unit to reduce density, and appropriate ventilation with ≥6 air changes per hour. Staffing enhancements included a temporary 1:1 nurse ratio for cohort patients, a dedicated infection control practitioner presence during high-activity periods, dedicated nursing staff with no cross-coverage, and minimized use of float staff.

Education and communication strategies

Education and training included daily 15-minute huddles at shift start, hand hygiene auditing with immediate feedback targeting >90% compliance, daily PPE competency checks, parental education using written Arabic materials and demonstrations, and enhanced environmental services training. Communication measures included daily written reports to executive leadership, weekly updates to the Ministry of Health, daily staff huddles and email updates, individual family discussions, and transparent communication regarding the outbreak situation.

Staff survey

A voluntary, anonymous questionnaire was administered to NICU staff involved in the outbreak response, including nursing staff, physicians, respiratory therapists, and infection-control practitioners. The survey was distributed following completion of the 2023 outbreak response and aimed to assess communication, perceived effectiveness of the outbreak-management framework, and staff experience during implementation. As the survey was designed as a descriptive process-evaluation tool to assess acceptability and perceived impact rather than to test a prespecified hypothesis, no formal sample size calculation was performed. The achieved sample size and response rate are therefore reported descriptively.

Data and statistical analysis

Data were analyzed using SPSS (IBM SPSS Statistics for Windows, version 27.0, IBM Corp., Armonk, NY). Nonparametric and exact tests were applied due to the small sample size, including Fisher's exact test for categorical variables and the Mann-Whitney U test for continuous variables. Incidence density rate ratios were calculated using exact Poisson methods. Hand-hygiene compliance data, collected as repeated observations over time, were analyzed using generalized estimating equations (GEE) with a logit link and exchangeable correlation structure to account for within-period correlation and provide adjusted standard errors and confidence intervals [9,24]. Statistical analyses were interpreted with emphasis on effect size, direction, and process reliability rather than formal hypothesis testing, consistent with quality-improvement methodology [19,22].

## Results

Outbreak characteristics and patient profiles

Nine neonates were affected: seven in 2022 and two in 2023. Mean gestational age was 30.1 ± 3.2 vs 33.5 ± 2.1 weeks (Mann-Whitney U = 3.5, p = 0.14), and mean birth weight was 1.64 ± 0.72 vs 1.59 ± 0.25 kg (U = 6.0, p = 0.89). No differences in sex or device exposure were detected, although the analysis was underpowered to exclude clinically relevant imbalances. Given the small number of affected neonates, baseline comparisons are presented descriptively to provide clinical context rather than to infer statistical equivalence (Table 2).

**Table 2:** Comparison of Patient Characteristics. Continuous variables were compared using the Mann-Whitney U test; categorical variables were compared using Fisher’s exact test. Given the limited sample size (n=9), the study is underpowered to detect clinically meaningful differences, and non-significant p-values should not be interpreted as evidence of equivalence. †: Mann-Whitney U test was used for comparison of continuous variables; ‡: Fisher’s exact test was used for comparison of categorical variables.

Characteristic	2022 (n = 7)	2023 (n = 2)	Test Statistic	p-value
Gestational age (weeks), mean ± SD	30.1 ± 3.2	33.5 ± 2.1	U = 3.5†	0.14
Birth weight (kg), mean ± SD	1.64 ± 0.72	1.59 ± 0.25	U = 6.0†	0.89
Male sex	5 (71%)	2 (100%)	‡	1
Central venous catheter	6 (86%)	2 (100%)	‡	1
Mechanical ventilation	5 (71%)	1 (50%)	‡	1.00

Process and compliance measures

Process indicators showed improvement during the intervention period. Hand-hygiene compliance rose from 78.9% (95% CI 72.6-84.2) to 92.0% (95% CI 89.4-94.1), absolute improvement +13.1 pp (Wald χ² = 28.4, p< 0.001, GEE-adjusted). Environmental-hygiene scores increased from 78% ± 8% to 96% ± 3% (t = 4.73, p< 0.01). PPE compliance reached 100% (95% CI 95.9-100).

Outbreak incidence density was lower during the intervention period, decreasing from 8.9 cases per 1,000 patient-days (95% CI 3.9-17.6) to 2.5 per 1,000 patient-days (95% CI 0.3-9.1) (exact Poisson rate ratio 0.28; 95% CI 0.06-1.24; p = 0.09). Duration reduced from 53 to 14 days (−74%) (Table 3).

**Table 3:** Process Measures and Incidence Density Comparison. §: Generalized estimating equations (GEE) with exchangeable correlation structure; ‖: Independent samples t-test; ¶: Exact Poisson rate ratio (95% CI 0.06-1.24); pp: percentage points, used to denote absolute differences between proportions.

Measure	2022 Baseline	2023 Intervention	Difference	Test Statistic	p-value	95% CI
Hand-hygiene compliance (%)	78.9 (72.6-84.2)	92.0 (89.4-94.1)	+13.1 pp	Wald χ² = 28.4§	<0.001	(7.3-18.9)
Environmental-hygiene score (%)	78 ± 8	96 ± 3	+18 pp	t = 4.73‖	<0.01	(9-27)
PPE compliance (%)	-	100 (95.9-100)	-		-	-
Incidence density (cases/1,000 pt-days)	8.9 (3.9-17.6)	2.5 (0.3-9.1)	−6.4	Rate ratio = 0.28¶	0.09	(−14.8 to 0.8)

 Staff survey

Thirty-four staff members completed the questionnaire (response rate 79%). Reported communication satisfaction increased from 40% to 88%, and 91% of respondents reported increased confidence in outbreak management. However, 68% reported increased fatigue, and 29% raised concerns regarding long-term sustainability.

## Discussion

This quality-improvement evaluation suggests that implementation of a structured outbreak-management framework incorporating early warning, multidisciplinary coordination, and continuous auditing was temporally associated with improvements in infection-control process measures in a resource-limited NICU. The framework coincided with a shorter observed outbreak duration and a numerically lower outbreak incidence density, with observed gains in process indicators. These findings support the feasibility of translating improvement science principles, such as PDSA cycles and real-time feedback, into outbreak settings where reactive approaches traditionally predominate [19-21].

Comparable studies from Europe and Asia demonstrate that effective control of *Serratia marcescens* and other MDRO outbreaks relies on early detection, multidisciplinary escalation, and sustained adherence to multimodal infection-prevention bundles [3,10]. Similar to these reports, the framework emphasized situational awareness and communication, two domains often neglected in conventional reactive responses [7,25]. By formalizing early-warning triggers and linking them to an institutional response protocol, the model helped bridge the gap between surveillance data and operational action, an area where many resource-limited units struggle due to infrastructure and coordination challenges [8].

The measurable gains in hand hygiene and environmental-cleaning compliance reflect both behavioral and system-level change. Embedding daily audits with immediate feedback fosters accountability and continuous learning [26,27], supporting the view that iterative feedback loops are critical for sustained improvement in infection prevention practices [19,21].

Although the rate-ratio reduction did not reach conventional statistical significance (p = 0.09), the magnitude and direction of effect and consistency across process indicators suggest a potentially meaningful clinical association. These observations are consistent with prior evidence suggesting that bundled infection-prevention approaches may be associated with improved neonatal infection-control performance when implemented through structured team-based models [9,24]. Given the small case series design, the study was not powered to detect modest changes in incidence; therefore, findings should be interpreted as descriptive and hypothesis-generating rather than confirmatory.

From a practical perspective, this framework suggests that structured outbreak response is achievable without major new resources. Most interventions-enhanced spacing, nurse assignment reorganization, and targeted education leveraged existing personnel and infrastructure, aligning with global IPC guidance emphasizing leadership, organizational culture, and management structures in outbreak readiness [7,20]. However, sustainability emerged as a concern, with staff reporting fatigue and resource strain, consistent with challenges described in other outbreak and improvement initiatives [20,21].

Several limitations should be recognized. The small sample size and slightly higher gestational age of affected infants in 2023, together with increased surveillance intensity, could confound results. Furthermore, the Hawthorne effect may have contributed to transiently improved compliance. Molecular typing also was not available, precluding confirmation of clonal relatedness among isolates, a limitation commonly encountered in resource-limited settings [6]. The follow-up period after outbreak resolution was relatively short; therefore, the absence of recurrence should be interpreted as supportive rather than causal evidence of sustained effectiveness [7].

Future work should focus on multicentre validation, integration of molecular epidemiology for strain tracking where feasible, longer follow-up, and formal cost-benefit evaluation to quantify return on investment [6,20]. Incorporating QI-based outbreak frameworks into neonatal preparedness standards could yield gains in safety culture, resilience, and antimicrobial stewardship.

## Conclusions

This quality-improvement evaluation suggests that transitioning from a conventional reactive infection-control approach to a structured, proactive PDSA-based framework was associated with shorter outbreak duration, lower outbreak intensity, and improved process compliance in a resource-constrained NICU. The findings highlight the practical value of early-warning triggers, multidisciplinary coordination, standardized response bundles, and real-time audit with feedback in strengthening outbreak management. While the small sample size and single-centre pre-post design limit generalizability and preclude causal inference, the framework proved feasible, rapidly deployable, and acceptable to staff, albeit with recognized workload and sustainability challenges. These results support consideration of integrating structured quality-improvement methodologies into neonatal outbreak preparedness and suggest that proactive, system-level approaches may enhance resilience and response effectiveness in similar clinical settings.
